# Supplementary material for: Population-scale proteome variation in human induced pluripotent stem cells
Source: eLife. 2020 Aug 10;9:e57390. doi: 10.7554/eLife.57390 (PMC7447446; doi:10.7554/eLife.57390)
Supplement: Supplementary file 1. — To facilitate comparison with other datasets we report here the number of proteins and peptides at FDR 1%. [file elife-57390-supp1.docx]

| Study | Cell/tissue type | Technology | Number of peptides | FDR info | Number of protein groups |
| --- | --- | --- | --- | --- | --- |
| This study | iPSC | TMT | 236,625 | 1% | 16,345 |
| Bekker-Jensen et al, 2017 | HeLa | Label Free | 744,887 | 1% | 14,200 |
| Kim et al, 2014 | 30 organs and cell types | Multiple | 293,700 | 1% | 30,057 |
| Branca et al, 2014 | A431 | iTRAQ | 77,785 | 1% | 13,078 |
| Wilhelm et al, 2014 | 60 tissues,  13 body fluids,  147 cell lines | Multiple | 598,845 | 3% on peptides, no FDR on proteins | 18,097 genes |
| Zhang et al, 2016 | 174 ovarian tumors | iTRAQ | NA | 1% | 9,600 (3 unique peptides) |

**Supp. Table 2 Comparison of proteome coverage across human proteomics datasets.** To enable comparison with other datasets we report here the number of proteins and peptides at FDR 1%.
