# Supplementary material for: Population-scale proteome variation in human induced pluripotent stem cells
Source: eLife. 2020 Aug 10;9:e57390. doi: 10.7554/eLife.57390 (PMC7447446; doi:10.7554/eLife.57390)
Supplement: Supplementary file 2. — Shown are the number of lines and donors for which matched mRNA and protein data are available. [file elife-57390-supp2.docx]

| **Disease status** | **Lines** | **Donors** |
| --- | --- | --- |
| Normal | 112 | 83 |
| Monogenic diabetes | 38 | 30 |
| Bardet-Biedl syndrome | 38 | 28 |
| Usher syndrome and congenital eye defects | 6 | 4 |
| Hereditary cerebellar ataxia | 4 | 3 |
| Kabuki syndrome | 3 | 2 |
| Congenital hyperinsulinism | 1 | 1 |

**Supp. Table 5 Disease status.** Shown are the number of lines and donors for which matched mRNA and protein data is available.
